# Omitting surgery in esophageal cancer patients with complete response after neoadjuvant chemoradiotherapy: a systematic review and meta-analysis

**DOI:** 10.1186/s13014-021-01947-7

**Published:** 2021-11-14

**Authors:** Jaehyeon Park, Ji Woon Yea, Se An Oh, Jae Won Park

**Affiliations:** Department of Radiation Oncology, Yeungnam University College of Medicine, 170, Hyeonchung-ro, Nam-gu, Daegu, 705-717 South Korea

**Keywords:** Esophageal cancer, Meta-analysis, Complete response, Neoadjuvant chemoradiotherapy

## Abstract

**Background:**

Neoadjuvant chemoradiotherapy (nCRT) followed by surgery is a standard treatment modality for locally-advanced esophageal cancer. However, patients who achieve clinical complete response (cCR) after nCRT have been reported to have better prognosis. Further, the role of surgery in these patients is controversial. Thus, this meta-analysis aimed to evaluate whether surgery is still useful in patients with cCR after nCRT.

**Methods:**

We systematically reviewed the MEDLINE, PubMed, Embase, Cochrane library, and Scopus databases for studies on surgical efficacy in complete responders after concurrent chemoradiotherapy for esophageal cancer. The publication date was set to January 1, 2010–January 31, 2020. The hazard ratio (HR) and risk ratio were used to compare the 2-year overall survival (OS), disease-free survival (DFS), incidence of locoregional failure, distant metastasis, and treatment mortality between the nCRT and nCRT plus surgery groups.

**Results:**

Six articles involving 609 patients were included. There was a significant benefit of nCRT for OS (HR = 0.80, 95% confidence interval [CI] 0.64–0.99, *p* = 0.04), but not for DFS (HR = 1.55, 95% CI 0.35–6.86, *p* = 0.56). The nCRT group tended to have lower mortality than the nCRT plus surgery group (risk ratio = 0.15, 95% CI 0.02–1.18, *p* = 0.07).

**Conclusion:**

Omitting surgery provides better OS in complete responders after nCRT. Adding surgery could increase the morbidity and mortality and decrease the quality of life. Thus, nCRT alone could be a feasible approach for patients with cCR.

**Supplementary Information:**

The online version contains supplementary material available at 10.1186/s13014-021-01947-7.

## Background

The International Agency for Research on Cancer estimates that 450,000 cases of esophageal cancer occurred in 2012 [1]. Esophageal cancer is the sixth leading cause of cancer-related mortality worldwide, and the 5-year survival rate is only approximately 20% [2, 3]. Regional differences in incidence and mortality are remarkable, with high incidence and mortality in Eastern Asia [2]. Surgery is the main curative modality for early stage esophageal cancer. However, most patients are diagnosed at the advanced stage [3]. For locally advanced operable disease, neoadjuvant chemoradiotherapy (nCRT) followed by surgery is recommended as a standard treatment based on the CROSS trial, which showed that nCRT followed by surgery significantly prolonged survival compared to surgery alone [4]. Chemoradiotherapy (CRT) alone is also considered a reasonable option for inoperable patients or those who refuse surgery. Some studies have shown no significant difference in survival between nCRT followed by surgery and CRT in locally advanced disease [5–7]. However, nCRT followed by surgery remains the preferred treatment for locally advanced operable disease due to better local control.

Several studies have argued that the wait-and-see approach is also feasible if there is complete remission after nCRT in locally advanced rectal cancer, which had a similar treatment approach with esophageal cancer [8, 9]. In esophageal cancer, complete remission after nCRT is also known to be a good prognostic factor, with patients in complete remission reported to have lower local recurrence and better survival than those with partial response and non-response [10, 11]. In the CROSS trial, the rate of pathological complete remission was 29% [49% for squamous cell carcinoma (SCC) and 23% for adenocarcinoma (ACC)] [4]. Therefore, considering the morbidity and mortality, the value of surgery in patients who have shown complete remission after nCRT is debatable.

A recent prospective study on the role of surgery in patients with complete remission after nCRT [12] has shown no significant difference in survival and locoregional failure (LF) rates between the surgery and non-surgery groups. The authors concluded that close observation with salvage surgery could be a reasonable choice in patients who achieved complete remission after nCRT. However, the number of enrolled patients was very small, and the conclusions about the role of surgery are still unclear. Thus, this systematic review and meta-analysis aimed to evaluate the usefulness of surgery in patients with complete remission after nCRT.

## Methods

### Search strategy and study selection

A systematic literature search was performed using the MEDLINE, PubMed, Embase, Cochrane library, and Scopus databases. We considered all studies on the therapeutic value of surgery in complete responders after concurrent CRT for esophageal cancer. The publishing date was set to between January 1, 2010 and January 31, 2020. The following terms were used for search: (esophageal OR esophagus OR oesophageal) AND (carcinoma OR neoplasm OR tumor) AND (chemoradiation OR chemoradiotherapy OR radiochemotherapy OR chemo-irradiation OR chemo-radiotherapy) AND (complete OR response OR responder OR complete response OR complete responder). The inclusion criteria were as follows: (1) studies on patients with esophageal cancer treated with concurrent CRT; (2) studies on patients who achieved clinical complete remission after concurrent CRT; and (3) studies comparing treatment outcomes between patients with esophageal cancer treated with and without surgery. The exclusion criteria were: (1) comparing radiotherapy or CRT with surgery alone; (2) reviews or case reports, with other sites of cancers, and meta-analysis; (3) unclear results; and (4) studies in written in language other than English.

### Quality assessment

The quality of the included studies was independently assessed by two investigators according to the type of the study. The Cochrane Collaboration’s tool was adopted for assessment of the randomized controlled trial. The quality evaluation included: method of randomization, allocation concealment, blinding, integrity of result data, results of selective reporting, and other sources of bias. Each element was qualified as high, low, or unclear risk of bias. The Newcastle–Ottawa Quality Assessment Scale for case–control studies was used to assess non-randomized studies. High quality was defined as 6–7 low risk of bias and score 7–10; moderate quality, 3–5 low risk of bias and score 4–6; and low quality, less than 3 risk of bias and score less than 4 (Additional file 3: Fig. S1; Additional file 1: Table S1). Disagreements were resolved by consensus between two authors.

### Data extraction and synthesis

After duplicate publications were deleted, two authors (J.P and J.W.P) independently evaluated potentially eligible studies that were identified by our search. Articles were screened for eligibility based on a review of the title and abstract. Then, the full text of eligible articles was accessed and read independently by two authors (J.P and J.W.P). Next, relevant data were extracted from the eligible studies. These included the first author, country, year of publication, study period, type of study, sample size, age, chemotherapy regimens, and radiation dose. Treatment outcome data included the 2-year overall survival (OS), disease-free survival (DFS), incidence of LF, distant metastasis (DM), and mortality. Patients who showed both LF and DM were classified in the DM group. If the hazard ratio (HR) and 95% confidence interval (CI) were not available, an estimate value was calculated by using the methods described by Tierney et al. [13]. Survival rates from Kaplan–Meier curves were read using Graph Grabber version 2.0.2 (Quintessa Ltd, England), and the resulting data were then entered in the calculation spreadsheet appended to Tierney’s paper.

### Statistical analyses

All analyses were performed using Review Manager Version 5.3. All statistical tests were two-sided. The relative risk (RR) or HR and its 95% CI was used to quantify the incidence of LF, DM, and treatment mortality, as well as the 2-year OS and DFS. When using the RR for evaluation, the significance was assessed using the Mantel–Haenszel test. In addition, inverse variance test was adopted when the HR was estimated. Q tests and I^2^ tests were adopted to estimate heterogeneity. Publication bias was evaluated using a funnel plot.

## Results

### Study characteristics

**Fig. 1:**
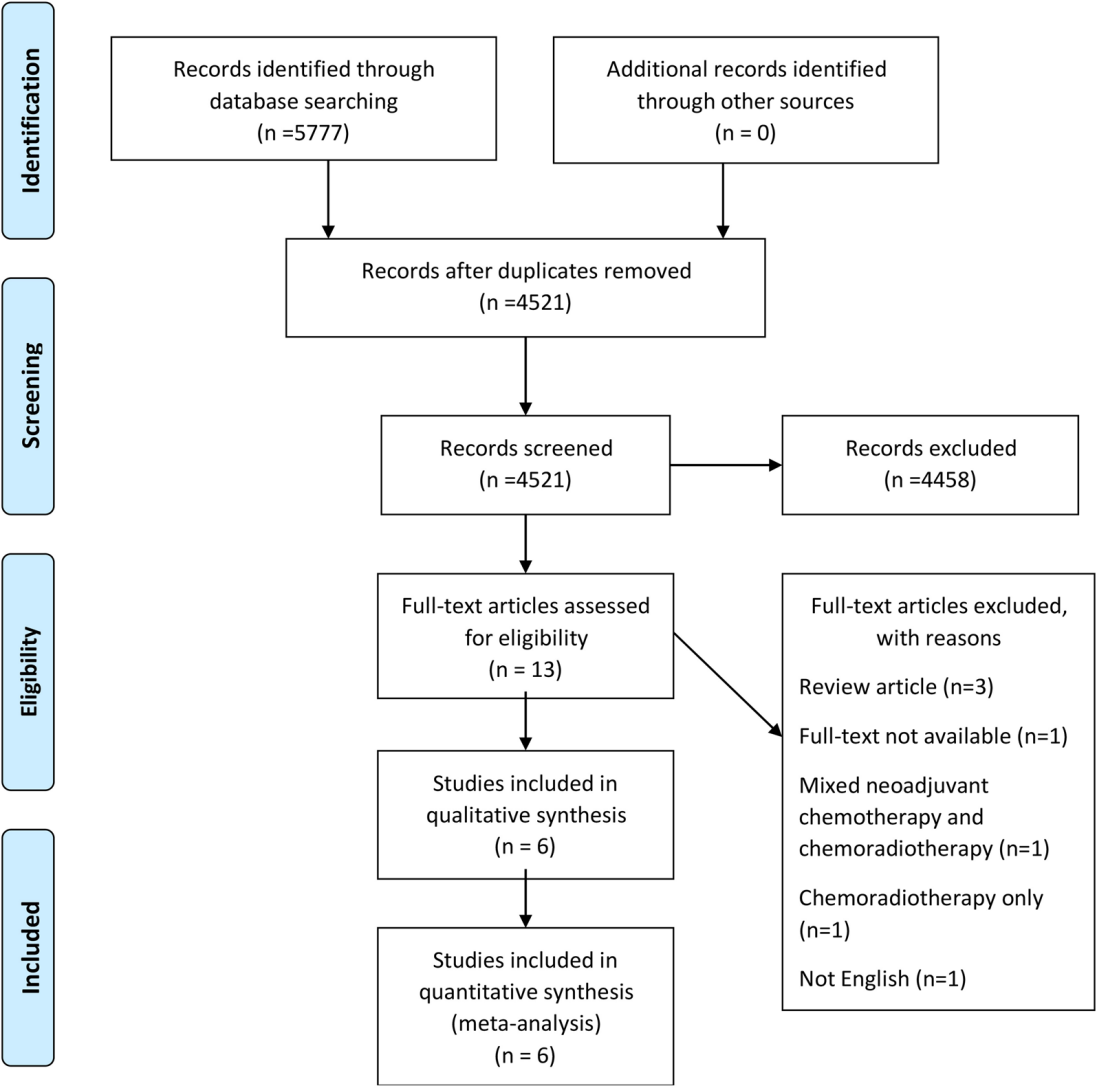
PRISMA flow diagram

Of the 5777 articles identified, 1256 duplicates were removed. After title and abstract screening, 13 articles were evaluated for their full text. Finally, six articles were included in the analyses (Fig. 1). The detailed characteristics of these included studies are summarized in Table 1. Only one study was a randomized prospective study, and the rest were retrospective studies. The studies were published in 2013–2019 and included a total of 609 patients who had clinical complete response (cCR) after nCRT. Of them, 353 and 256 patients were categorized to the nCRT plus surgery and nCRT groups, respectively. By histology, 509, 99, and one patient(s) had SCC, ACC, and adenosquamous carcinoma, respectively.

**Table 1:** Characteristics of the included studies

Author (study period)	Nation	Study design	No. of patients	Histology	Clinical stage	Neoadjuvant chemoradiotherapy (nCRT)	Work-up after nCRT	Outcome (2 yr-OS (%)/2 yr-DFS (%))	Quality assessment^b^
Castro [15] (1992–2007)	Italy	Retrospective	nCRT (n = 38)	SCC only	II–IV	nCRT 45–50 Gy in 1.8 Gy daily fraction with FP	Endoscopy with biopsies, CT	72.2/39.8	Moderate
			nCRT + S (n = 39)					72.2/64.8	
Chao [16] (1996–2006)	Taiwan	Retrospective	nCRT (n = 79)	SCC only	II–IV^a^	Induction FP#1 + nCRT 30 Gy/15 fx with FP boost 30 Gy/15fx (pT3 or pN + in in surgery or in without surgery)	Endoscopy with biopsies, CT	62.8	Moderate
			nCRT + S (n = 71)					56.1	
Piessen [17] (1995–2012)	France	Retrospective	nCRT (n = 59)	SCC (n = 149)	II–III	nCRT 45 Gy/25 fx with FP	Endoscopy with biopsies, barium swallow, CT (PET was used selectively.)	58.0/58.2	High
			nCRT + S (n = 118)	ADC (n = 28)				81.0*/80.4*	
Jeong [14] (2005–2008)	Korea	Retrospective	nCRT (n = 31)	SCC only	II–IV^a^	Induction XP#2 + nCRT with XP	Endoscopy with or without biopsy, EUS, CT, PET	61.3/47.3	Moderate
			nCRT + S (n = 39)			nCRT + S: 46 Gy/23 fx		71.8/83.0*	
						nCRT: 54 Gy/27 fx			
Wilk [18] (2013–2016)	Netherland	Retrospective	nCRT (n = 29)	SCC (n = 26)	II–III	nCRT 41.4 Gy/23 fx with PC	Endoscopy with biopsies (bite-on-bite), EUS, PET	89.7/73.5	High
				ADC (n = 71)					
				ADSC (n = 1)					
			nCRT + S (n = 29)					69.0/76.3	
Park [12] (2012–2016)	Korea	Prospective	nCRT (n = 18)	SCC only	II–III	Induction XP#2 + nCRT with XP	Endoscopy with or without biopsy, EUS, CT, PET	72.8/42.7	Moderate
			nCRT + S (n = 19)			50.4 Gy/28 fx with XP		74.4/66.7	

*nCRT* neoadjuvant chemoradiotherapy, *nCRT + surgery* neoadjuvant chemoradiotherapy followed by surgery, *SCC* squamous cell carcinoma, *ADC* adenocarcinoma, *ADSC* adenosquamous cell carcinoma, *CT* computed tomography, *PET* positron emission tomography, *EUS* endoscopic ultrasonography, *FP* 5-fluorouracil/cisplatin, *XP* cisplatin/capecitabine, *PC* carboplatin/paclitaxel, *OS* overall survival, *DFS* disease-free survival

^a^Distant metastasis, lymph node metastasis other than regional LN

^b^Assessed using the Newcastle–Ottawa Quality Assessment Scale for retrospective studies and The Cochrane Collaboration’s tool for prospective studies

*Statistically significant

Three studies were from Asia (two from South Korea and one from Taiwan), and the other three were from Western countries (one each from France, Italy, and the Netherlands). Induction chemotherapy followed by nCRT was performed in three studies, while nCRT was administered before surgery in the other three studies. The total delivered radiation dose ranged from 30 Gy from 60 Gy. Two studies [12, 14] reported the HR and 95% CI of OS and DFS. Those of the remaining four studies [15–18] were estimated based on the Kaplan–Meier curves using the method mentioned above.

### Overall survival and disease-free survival

**Fig. 2:**
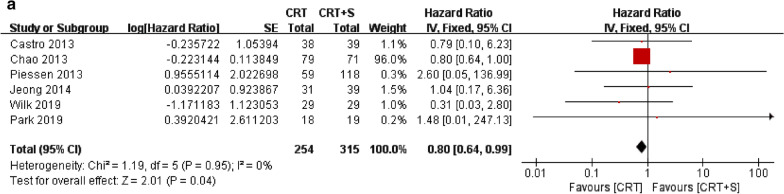

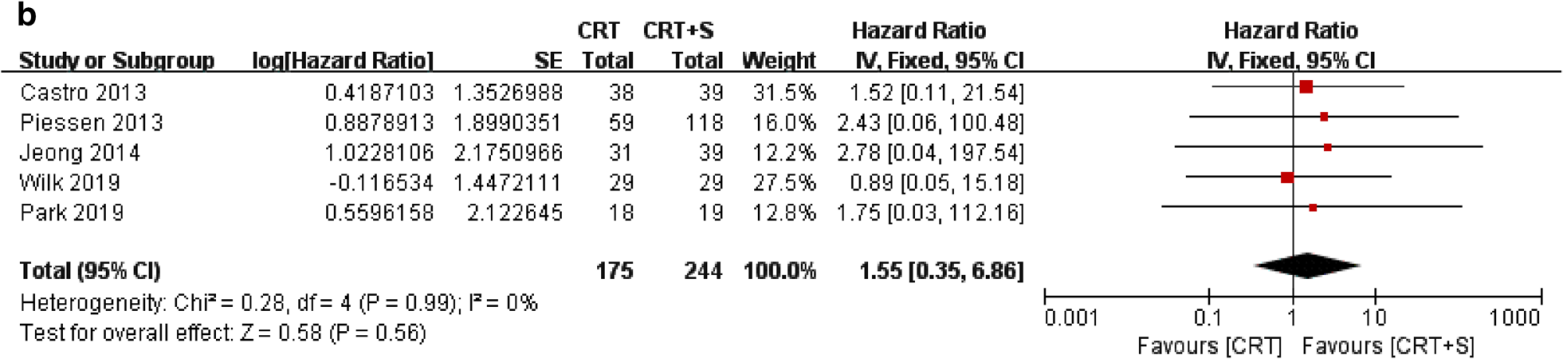
Forest plot of comparison of **a** overall survival (OS) and **b** disease-free survival (DFS)

For OS assessment, there was no significant heterogeneity for the results among the studies (*p* = 0.95, I^2^ = 0%); thus, a fixed-effects model was used for further analysis. As shown in Fig. 2a, patients in the nCRT group had significantly better OS than those in the nCRT plus surgery group (HR = 0.80, 95% CI 0.64–0.99, *p* = 0.04).

For DFS assessment, there was also no significant heterogeneity for the results among the studies (*p* = 0.99, I^2^ = 0%); thus a fixed-effects model was used for further analysis. In contrast to OS, we found no significant difference in DFS between the nCRT and nCRT plus surgery groups (HR = 1.55, 95% CI 0.35–6.86, *p* = 0.56; Fig. 2b).

### Patterns of failure and treatment mortality

**Fig. 3:**
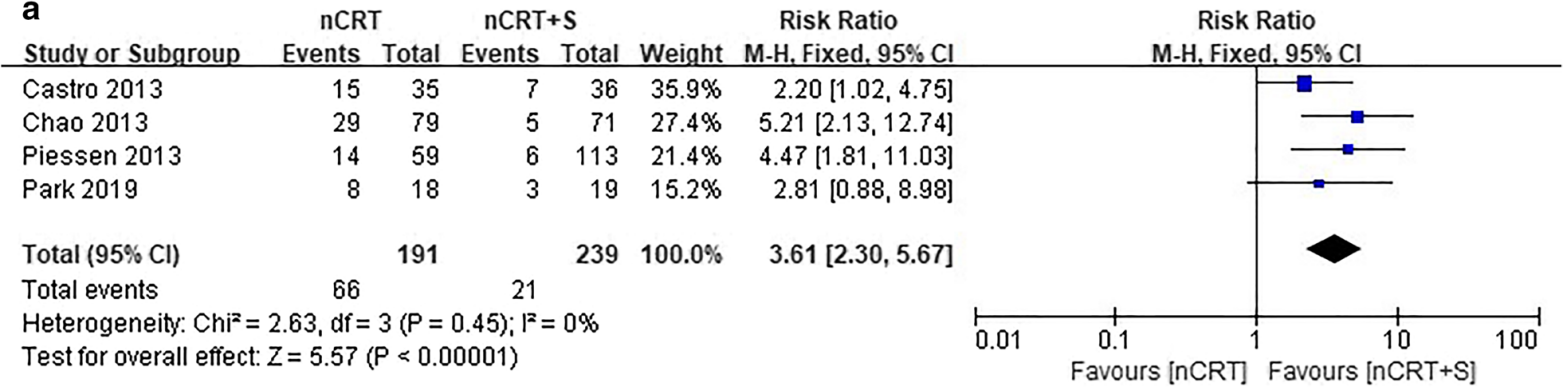

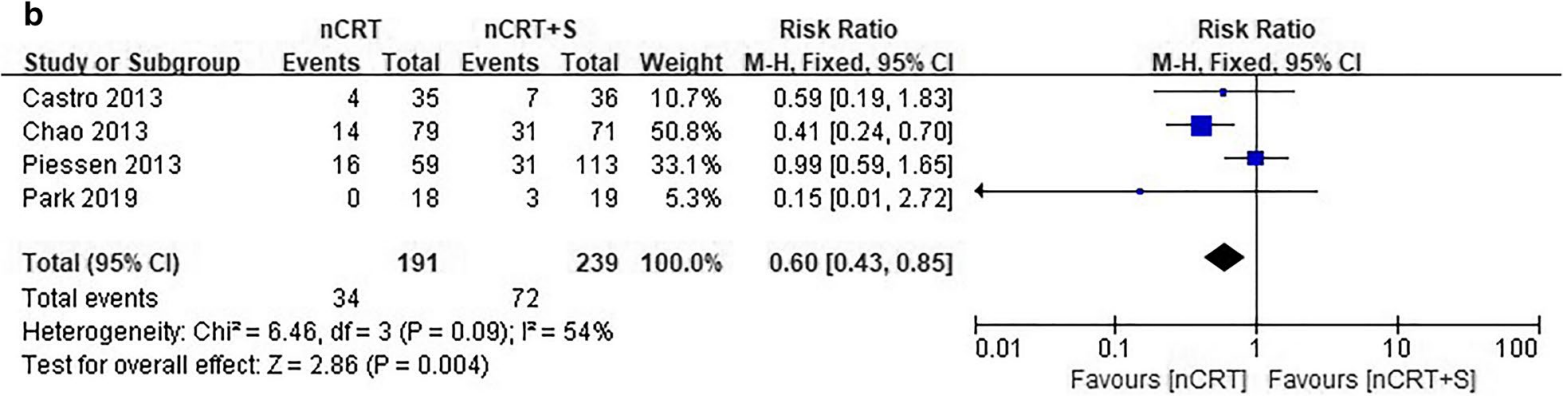
Forest plot of patterns of failure. **a** locoregional failure and **b** distant failure

Data on failure rate according to recurrence type were available in four studies. The incidence rate of locoregional recurrence (LR) was significantly lower in the nCRT plus surgery group than in the nCRT group (RR = 3.61, 95% CI 2.30–5.67, *p* < 0.001). Meanwhile, the nCRT group had a significantly lower DM rate than the nCRT plus surgery group (RR = 0.60, 95% CI 0.43–0.85, *p* = 0.004; Fig. 3).

**Fig. 4:**
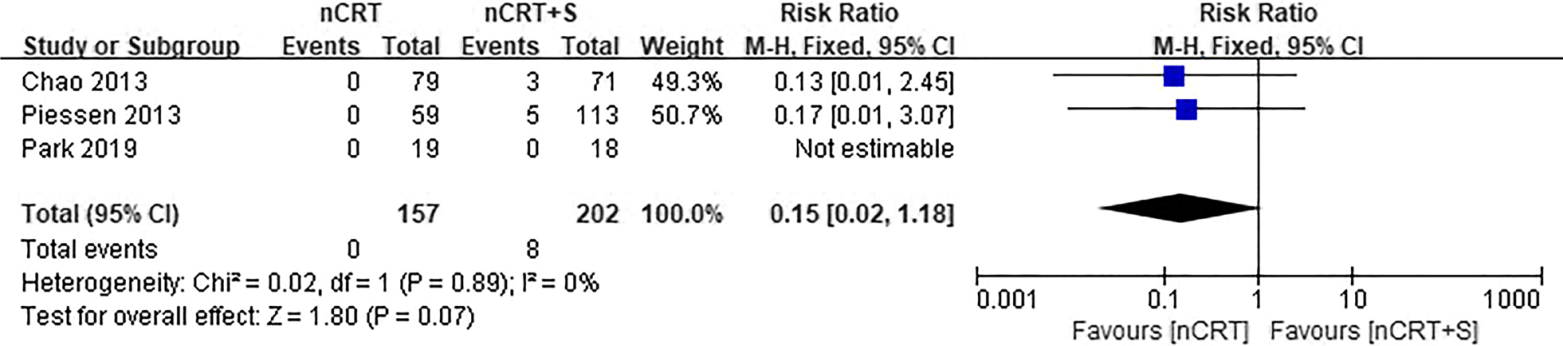
Forest plot of comparison of treatment mortality

Only three studies reported on treatment mortality (Fig. 4). The nCRT group tended to have lower mortality than the nCRT plus surgery group, although the difference was not statistically significant (RR = 0.15, 95% CI 0.02–1.18, *p* = 0.07).

### Sensitivity analysis and publication bias

Sensitivity analysis was applied by sequentially removing each study, and the results were stable (Additional file 2: Table S2). Publication bias statistical analysis performed using the funnel plot also showed that no publication bias exists in the meta-analysis (Additional file 4: Fig. S2).

## Discussion

The role of surgery in patients who achieve cCR after nCRT is controversial. This systematic review and meta-analysis showed that nCRT alone has better survival benefit than nCRT plus surgery. There was no significant difference in DFS (HR = 1.55, 95% CI 0.35–6.86). This survival benefit in nCRT may be attributed to the lower mortality in the nCRT group than in the nCRT plus surgery group and the pattern of failure between nCRT and nCRT plus surgery. Specifically, nCRT plus surgery showed better local control, although it also showed a higher incidence of distant failure than nCRT.

Currently, nCRT plus surgery is the preferred approach for locally advanced operable esophageal cancer. One study showed that salvage esophagectomy provides patients with LR a chance for long-term survival [19]. CRT alone is also a feasible option for these patients, and several studies showed no benefit of the addition of surgery [5–7]. Thus, it raises the question of whether surgery is of benefit in locally advanced operable esophageal cancer. Particularly, in the case of complete responders after nCRT, who are known to have low recurrence rates and high survival rates [20], omitting surgery could be more beneficial because it avoids the morbidity and mortality associated with the addition of surgery. Wilk et al. retrospectively compared the treatment outcomes between active surveillance and immediate surgery in patients with cCR after nCRT [18] and found that although there was no statistical difference, the survival rates were higher in the active surveillance group (3-year OS = 77% vs. 55%, *p* = 0.10; 3-year progression-free survival [PFS] = 60% vs. 54%, *p* = 0.87). Meanwhile, a recent prospective study showed that nCRT is associated with a higher relapse rate and a lower survival rate (HR = 1.17, *p* = 0.28 for PFS; HR = 1.48, *p* = 0.56 for OS) [12]. The cCR rate was 47.6%, which is approximately 7% higher than that reported in Wilk’ study. However, the two studies differed in the assessment of cCR, as the former used a bite-on-bite biopsy and the latter a regular biopsy. The recently published preSANO study revealed that compared with regular biopsies, the sensitivity for the detection of residual disease increased substantially from 54 to 74% with bite-on-bite biopsies [21]. We could presume that inclusion of more patients with false-negative results could cause a detrimental effect on the analysis of treatment outcomes of nCRT.

Thus, the important issue is the accuracy of cCR for predicting pathologic CR (pCR) and its reliability as an indicator for omitting surgery in cCR after nCRT in esophageal cancer. Endoscopy or endoscopic ultrasonography (EUS) with biopsy, computed tomography (CT), and positron emission tomography (PET)-CT are generally used to evaluate treatment response after nCRT. However, no single modality could produce satisfactory results. A meta-analysis on the accuracy of modalities showed pooled sensitivities and specificities of respectively 33% and 95% for endoscopic biopsies, 96% and 8% for qualitative EUS, 74% and 52% for qualitative PET, 69% and 72% for PET using the maximum standardized uptake value (SUV_max_), and 73% and 63% for PET using percentage reduction of SUV_max_ [22]. A systematic study reported that the negative predictive value of negative endoscopy biopsy after CRT was only 47% [23]. Cheedella et al. reported the result of response evaluation after CRT using endoscopy with biopsy and the SUV_max_ of PET-CT [24]. The sensitivity of cCR for pCR was 97.1%, and the specificity was 29.8%. They concluded that cCR is not highly associated with pCR due to the low specificity. However, magnetic resonance imaging (MRI) is emerging as a promising method for response evaluation as it was highly predictive of histopathologic response and has the potential benefit of a multiparametric approach using diffusion-weighted and dynamic contrast-enhanced imaging [25, 26]. A recent prospective study reported that PET and MRI are effective in predicting pathologic response [27]. In addition, this study reported that a combined model of MRI, PET, and histology could improve the predictive rate compared with using each modality alone (c-statistic 0.84 vs. 0.79).

There are ongoing efforts to identify factors that can predict the response of esophageal cancer by neoadjuvant treatment. Sherry et al. showed that dynamic time-dependent changes in the neutrophil-to-lymphocyte ratio (NLR) during nCRT predict response and clinical outcomes [28]. Increasing NLR was associated with a reduced probability of pCR (odds ratio = 0.80, *p* = 0.03), shortened DFS (HR = 1.02, *p* < 0.01), and reduced OS (HR = 1.02, *p* < 0.01). Maher et al. reported that good responders to nCRT can be predicted with low pretreatment complement C3a and C4 [29]. As cancer genetics research progresses, both biochemical and molecular predictive factors have been proposed. P53 (a well-known tumor suppressor gene) and excision-repair cross-contemplating 1 (ERCC1) (an important enzyme in the nucleotide excision repair pathway), are representative. According to a meta-analysis, wild-type p53 had a high pCR rate to nCRT in SCC (RR = 1.13, *p* = 0.04) [30]. Also, the low level of ERCC1 mRNA expression is associated with a superior response to platinum-based chemotherapy in SCC [31]. Metager et al. showed that ERCC1 (rs11615) gene polymorphisms are associated with response and survival in patients with ACC who underwent nCRT [32].

Surgery has a major impact on the patient’s quality of life (QOL). Boehier et al. investigated long-term QOL and symptom evolution up to 20 years after esophagectomy [33] and found that esophagectomy was associated with decreased QOL and lasting gastrointestinal symptoms up to 20 years after surgery. Meanwhile, CRT negatively affects the QOL during treatment, although it mostly recovered afterwards. Noordman et al. reported that a negative impact of nCRT on QOL was observed only during the last cycle of CRT and up to 2 weeks after CRT [34]. Physical functioning, odynophagia, and sensory symptoms were restored to baseline levels from 1 to 2 months after nCRT. Odynophagia, fatigue, and weight loss improved after nCRT within 4 months.

In this study, the risk of treatment mortality was lower in nCRT than in nCRT plus surgery. However, this does not include mortality after salvage surgery in cases of LR in nCRT. Several previous studies report that patients who receive salvage surgery have high morbidity and mortality, with anastomosis leakage occurring in 21–38% and the mortality ranging from 4%–33%. Meanwhile, survival was similar to that of patients who received planned surgery [35–39]. However, these results should be interpreted with caution due to the inclusion of inoperable patients who received high-dose radiotherapy (> 60 Gy). Notably, the morbidity and mortality have greatly reduced in the recent decade, and the differences of those between planned and salvage surgery have also reduced. In the MD Anderson Cancer Center (UICC), comparison between the early (1987–2000) and modern eras (1997–2010) showed that the mortality rate decreased from 6 to 3% for planned surgery and from 15 to 5% for salvage surgery [40]. Indeed, better survival in nCRT may reflect advancements in surgical techniques, lower radiotherapy doses, and selection of patient with cCR.

Recently, research on proton beam therapy (PBT), an emerging advanced radiotherapy technology, is being actively conducted for esophageal cancer. The esophagus is anatomically located in the midline and surrounded by critical organs (i.e., the heart and lungs). Featuring the highest dose at the Bragg peak and rapid dose fall off beyond that point [41], PBT can reduce the radiotherapy dose to these organs [42, 43]. Lin et al. retrospectively analyzed 580 patients with nCRT to investigate difference according to radiotherapy modality, including PBT, intensity modulated radiotherapy (IMRT), and three-dimensional conformal techniques (3D-CRT) [44]. The incidence of pulmonary complications of PBT, IMRT, and 3D-CRT was 16.2%, 24.2%, and 39.5% (*p* < 0.01), respectively; the incidence of cardiac complications was 11.7%, 11.7%, and 27.4%, respectively (*p* < 0.01). The non-statistically significant 90-day postoperative mortality (0.9%, 4.3%, and 4.2% in PBT, IMRT, and 3D-CRT, respectively) was the lowest in PBT (*p* = 0.26). The UICC retrospectively analyzed patients who underwent definitive CRT and showed significantly better 5-year OS (41.6% vs. 31.6%, *p* = 0.01); PFS (34.9% vs. 20.4%, *p* < 0.01); and distant metastasis-free survival (DMFS) (64.9% vs 49.6%, *p* = 0.03) in the PBT group compared with the IMRT group [43]. In subgroup analysis, no significant difference occurred in the 5-year OS, PFS, DMFS, and locoregional failure-free survival (LRFFS) in stage I/II; however, in stage III, the 5-year OS and PFS in the PBT group were significantly higher (34.6% vs. 25.0%, *p* = 0.04; 33.5% vs. 13.2%, *p* < 0.01, respectively) and the LRFFS was also better (62.6% vs. 43.4%, *p* = 0.051). It is questionable how the improvement in OS and decrease in treatment toxicity, including postoperative mortality, by PBT will affect the prognosis of patients with CR after nCRT. It is expected that ongoing prospective randomized trials, such as the NRG-GI006 (NCT03801876) and European PROTECT trial (IMI 101008134), will provide clues to these questions [45, 46].

This study has some limitations that should be considered when interpreting the results. First, five of the six studies selected were retrospective. Thus, selection bias could be inevitable between nCRT and nCRT plus surgery, and cofounding factors cannot be balanced. Second, the radiotherapy doses used in the included studies were heterogeneous, ranging from 30to 50.4 Gy. A systematic analysis reported that higher radiation doses increased the probability of achieving pCR [47]. However, several studies showed no association between the radiotherapy dose and survival [48, 49]. Rather, a high dose may be associated with inferior OS due to increased mortality after surgery [50]. Although not significant in our study, high mortality following nCRT plus surgery may have influenced the favorable outcome for nCRT alone; thus, a further study is needed to understand the optimal radiation dose in nCRT. Third, the analysis of long-term outcomes included limited survival data of only up to 2 years. However, there may be little difference in the survival tendency because most recurrence occur within 2 years. Current large prospective studies, such as the SANO- and ESOSTRATE-trials, will resolve this limitation. Fourth, subgroup analyses of esophageal ACC and SCC could not be performed because all included studies did not provide treatment result according to histology. Most patients in this meta-analysis had SCC, which is generally known to be more sensitive to CRT. Although there is no significant difference in the complete response rates between ACC and SCC (median 22.0% [range, 9.0%–40.0%] vs. 23.7% (range, 16.0%–41.0%) [51], further research on the feasibility of omitting surgery after nCRT in ACC is needed. Further prospective randomized controlled clinical trials with large sample sizes are also needed to validate our findings.

## Conclusion

The findings of this systematic review and meta-analysis showed that nCRT alone provides better OS benefit than nCRT plus surgery in complete responders after nCRT. Adding surgery could increase the morbidity and mortality and decrease the QOL. Thus, nCRT without surgery could be a feasible approach for patients with cCR.

## Supplementary Information

**Additional file 1. Table S1**: Quality assessment of the included retrospective studies according to the Newcastle-Ottawa Scale (NOS).

**Additional file 2. Table S2**: Sensitive analysis: (A) overall survival, (B) disease-free survival, (C) locoregional failure, (D) distant failure, and (E) treatment mortality.

**Additional file 3. Fig. S1**: The Cochrane Collaboration’s tool for the quality assessment of prospective studies.

**Additional file 4. Fig. S2**: Funnel test for publication bias: (a) overall survival, (b) disease-free survival, (c) locoregional failure, (d) distant failure and (e) treatment mortality.

## Data Availability

All data are fully available without restriction.

## Abbreviations

nCRT: Neoadjuvant chemoradiotherapy
CRT: Chemoradiotherapy
LF: Local failure
OS: Overall survival
DFS: Disease-free survival
DM: Distant metastasis
cCR: Clinical complete response
RR: Relative risk
HR: Hazard ratio
CI: Confidence interval
SCC: Squamous cell carcinoma
ACC: Adenocarcinoma
LR: Locoregional recurrence
pCR: Pathologic CR
EUS: Endoscopic ultrasonography
CT: Computed tomography
PET: Positron emission tomography
MRI: Magnetic resonance imaging (MRI)
QOL: Quality of life
