# Supplementary material for: Omitting surgery in esophageal cancer patients with complete response after neoadjuvant chemoradiotherapy: a systematic review and meta-analysis
Source: Radiat Oncol. 2021 Nov 14;16:219. doi: 10.1186/s13014-021-01947-7 (PMC8591817; doi:10.1186/s13014-021-01947-7)
Supplement: Supplementary file 1 — Additional file 1. Table S1: Quality assessment of the included retrospective studies according to the Newcastle-Ottawa Scale (NOS). [file 13014_2021_1947_MOESM1_ESM.docx]

Supplement Table 1. Quality assessment of the included retrospective studies according to the Newcastle-Ottawa Scale (NOS)

| Study | Selection | Comparability | Expose |
| --- | --- | --- | --- |
| Castoro 2013 | ★★ | ★★ | ★★ |
| Chao 2013 | ★★ | ★★ | ★★ |
| Piessen 2013 | ★★★ | ★★ | ★★ |
| Jeong 2014 | ★★ | ★ | ★★ |
| Wilk 2019 | ★★★ | ★★ | ★★ |
