# Supplementary material for: Omitting surgery in esophageal cancer patients with complete response after neoadjuvant chemoradiotherapy: a systematic review and meta-analysis
Source: Radiat Oncol. 2021 Nov 14;16:219. doi: 10.1186/s13014-021-01947-7 (PMC8591817; doi:10.1186/s13014-021-01947-7)
Supplement: Supplementary file 2 — Additional file 2. Table S2: Sensitive analysis: (A) overall survival, (B) disease-free survival, (C) locoregional failure, (D) distant failure, and (E) treatment mortality. [file 13014_2021_1947_MOESM2_ESM.docx]

Supplement Table 2. Sensitive analysis: (A) overall survival, (B) disease-free survival, (C) locoregional failure, (D) distant failure, and (E) treatment mortality

(A) Overall survival

| Omitted study | Harzard ratio | 95% confidential interval |
| --- | --- | --- |
| Castoro 2013 | 0.80 | 0.64-0.99 |
| Chao 2013 | 0.75 | 0.26-2.17 |
| Piessen 2013 | 0.80 | 0.64-0.99 |
| Jeong 2014 | 0.80 | 0.64-0.99 |
| Wilk 2019 | 0.81 | 0.62-1.00 |
| Park 2019 | 0.80 | 0.64-0.99 |

(B) Disease-free survival

| Omitted study | Harzard ratio | 95% confidential interval |
| --- | --- | --- |
| Castro 2013 | 1.14 | 0.36-3.64 |
| Chao 2013 | 1.55 | 0.36-6.60 |
| Piessen 2013 | 1.12 | 0.37-3.40 |
| Jeong 2014 | 1.12 | 0.37-3.40 |
| Wilk 2019 | 1.25 | 0.40-3.95 |
| Park 2019 | 1.16 | 0.39-3.50 |

(C) Locoregional failure

| Omitted study | Harzard ratio | 95% confidential interval |
| --- | --- | --- |
| Castro 2013 | 4.39 | 2.51-7.70 |
| Chao 2013 | 3.00 | 1.79-5.03 |
| Piessen 2013 | 3.37 | 2.00-5.69 |
| Park 2019 | 3.75 | 2.30-6.12 |

(D) Distant failure

| Omitted study | Harzard ratio | 95% confidential interval |
| --- | --- | --- |
| Castro 2013 | 0.61 | 0.42-0.87 |
| Chao 2013 | 0.81 | 0.51-1.28 |
| Piessen 2013 | 0.41 | 0.26-0.67 |
| Park 2019 | 0.63 | 0.44-0.89 |

(E) Treatment mortality

| Omitted study | Harzard ratio | 95% confidential interval |
| --- | --- | --- |
| Chao 2013 | 0.17 | 0.01-3.07 |
| Piessen 2013 | 0.13 | 0.01-2.45 |
| Park 2019 | 0.15 | 0.02-1.18 |
