# Supplementary figures and images for: Omitting surgery in esophageal cancer patients with complete response after neoadjuvant chemoradiotherapy: a systematic review and meta-analysis
Source: Radiat Oncol. 2021 Nov 14;16:219. doi: 10.1186/s13014-021-01947-7 (PMC8591817; doi:10.1186/s13014-021-01947-7)

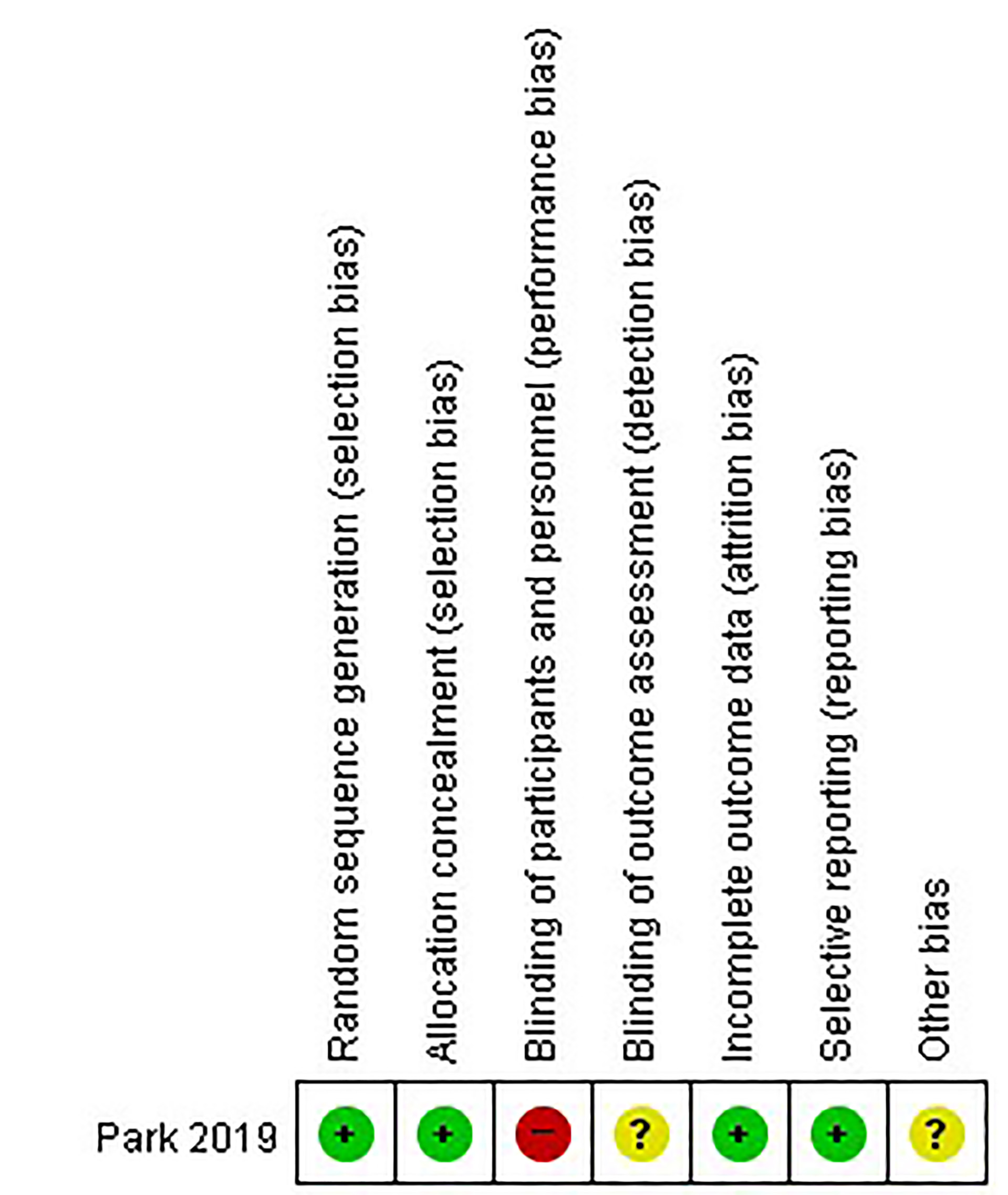

Supplement: Supplementary file 3 — Additional file 3. Fig. S1: The Cochrane Collaboration’s tool for the quality assessment of prospective studies. [file 13014_2021_1947_MOESM3_ESM.jpg]

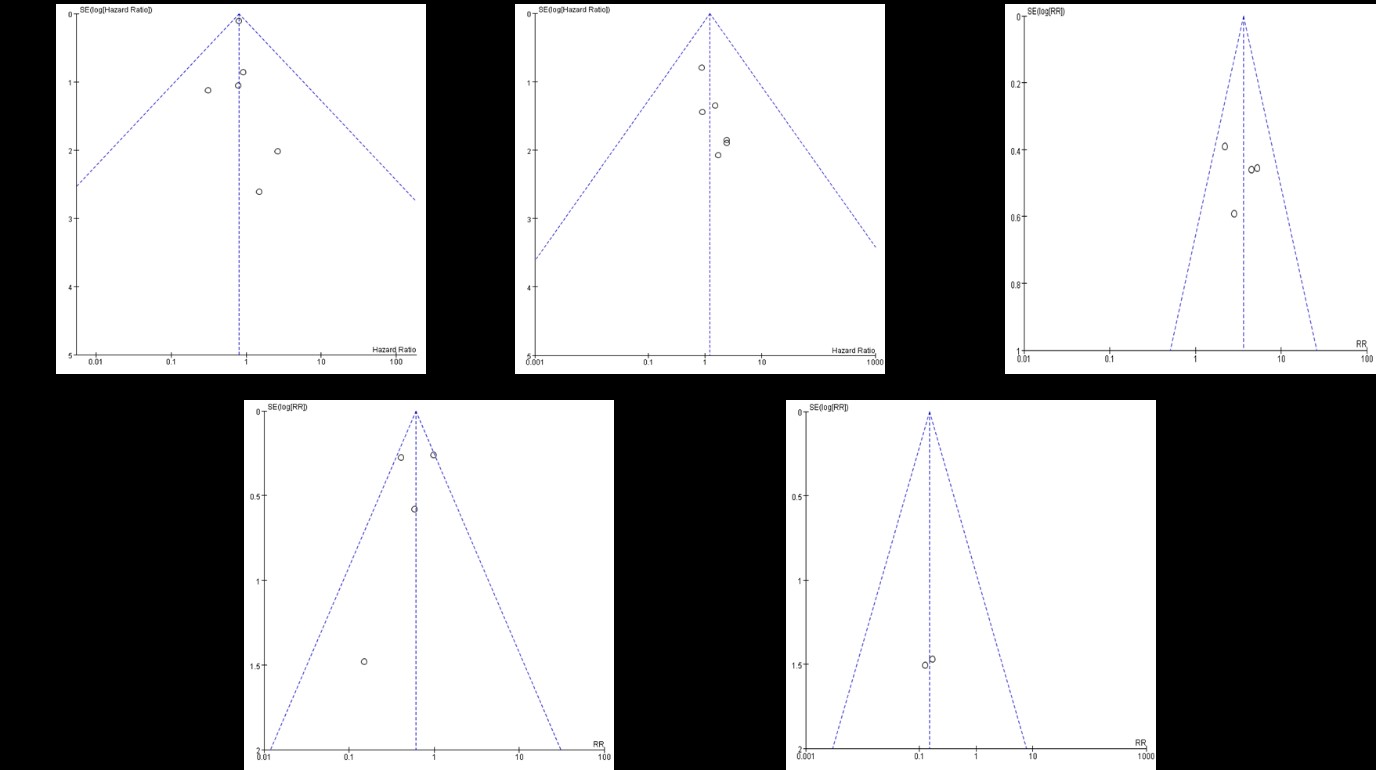

Supplement: Supplementary file 4 — Additional file 4. Fig. S2: Funnel test for publication bias: (a) overall survival, (b) disease-free survival, (c) locoregional failure, (d) distant failure and (e) treatment mortality. [file 13014_2021_1947_MOESM4_ESM.jpg]
